# Food Gels of Fish Protein Isolate from Atlantic Cod (*Gadus morhua*) By-Products Recovered by pH Shift

**DOI:** 10.3390/gels11120970

**Published:** 2025-12-02

**Authors:** Svetlana Derkach, Yuliya Kuchina, Daria Kolotova, Ekaterina Borovinskaya, Svetlana Voropaeva, Nikolay Voron’ko, Alena Nikiforova, Mariya Klimovitskaya, Alexander Klimovitskii, Vladislav Abramov, Elena Anikeenko, Yuriy Zuev

**Affiliations:** 1 Institute of Natural Sciences and Technology, Murmansk Arctic University, Sportivnaya St. 13, Murmansk 183010, Russia; kuchinayua@mauniver.ru (Y.K.); kolotovads@mauniver.ru (D.K.); borovinskayaev2@mauniver.ru (E.B.); voropaevaso@mauniver.ru (S.V.); voronkong@mauniver.ru (N.V.); 2 Kazan Institute of Biochemistry and Biophysics, FRC Kazan Scientific Center of RAS, Lobachevsky St. 2/31, Kazan 420111, Russia; alnikiforova22@gmail.com (A.N.); mklimovitskaya@mail.ru (M.K.); abramovv660@gmail.com (V.A.); yufzuev@mail.ru (Y.Z.); 3 A. Butlerov Chemical Institute, Kazan Federal University, Kremlevskaya St. 18, Kazan 420008, Russia; aklimovi@mail.ru; 4 Core Facility Center “Arktika”, Northern (Arctic) Federal University, Severnoy Dviny Nab. 17, Arkhangelsk 163002, Russia; a.anikeenko@narfu.ru

**Keywords:** food gels, fish protein isolate, alkaline solubilisation, isoelectric precipitation, functional properties, physico-chemical properties

## Abstract

Food gels were obtained using fish protein isolate recovered from Atlantic cod (*Gadus morhua*) by-products using the isoelectric solubilisation/precipitation method. The use of low temperatures (not exceeding 10 °C) at the alkaline solubilisation stage resulted in the production of a fish protein isolate with high-molecular mass (FPI-1), while the use of high temperatures (24 °C) resulted in the production of a fish protein isolate with low-molecular mass (FPI-2). The isolates demonstrated excellent gelling and nutritional properties based on their amino acid profiles. The denaturation temperatures of FPI-1 and FPI-2 determined by DSC were 163.0 and 158.5 °C. The secondary structure of FPI-1 demonstrated a high α-helix content and a low random coil content compared to FPI-2. The high-molecular isolate formed stronger gels than the low-molecular isolate, which is explained by the formation of a dense gel network with small pores of about 250 nm. The recovered cod protein isolates can be successfully used as food ingredients or food additives in the production of gel-like/enriched products.

## 1. Introduction

Among the variety of food products, a special place is occupied by products from marine bioresources, especially fish raw materials. In northern latitudes, one of the most important commercial products is Atlantic cod (*Gadus morhua*). Cod is popular as a food fish, characterised by its mild flavour and low content of fat. Currently, the volume of catch throughout the world in absolute terms is approximately 500,000 tons of Atlantic cod per year according to the Food and Agriculture Organization of the United Nations [1]. Russia and Norway lead the global catch of Atlantic cod, which inhabits northern seas. The chemical composition of Atlantic cod muscle, which determines its nutritional value and taste properties, is characterised by the contents of protein (15–19%), water (79–82%), fat (0.3–1.2%), and mineral substances and micronutrients (1.2–2.5%), as well as vitamins [2,3]. Fish proteins contain all essential amino acids [4], which is why they are in high demand as a complete protein food product.

Food waste generated by industrial enterprises during cod processing can make up to 30–70% of the total weight [5]. The volume of by-product increases even more as the demand for fish products in the form of fillets increases. Cod processing waste (skin, bones, scales, visceral organs, and muscle tissue trimmings) are valuable protein-containing raw materials and can be successfully used to obtain protein ingredients for new product development [6]. However, by-products from the fishing industry are often underutilised [7]. The use of secondary raw materials for industrial purposes simultaneously solves the problem of the complex (waste-free) processing of biological resources extracted in marine waters through traditional fishing and the environmental problem associated with industrial waste disposal.

One popular product made from secondary raw materials is fish protein isolate (FPI) [8], which preserves the properties of native fish proteins. Fish muscle tissue proteins, which include Atlantic cod muscle proteins, are divided into three groups depending on their participation in the construction of muscle tissue [9]. Myofibrillar proteins, which include myosin, actin, actomyosin and tropomyosin, are complete proteins and constitute the main portion of protein substances, with up to 55–65% of the total protein in muscles. Sarcoplasmic proteins, including globulin X, myogen, myoglobin, and myoalbumin, comprise 20–22% of the total protein content in muscle tissue, on average. Stromal proteins (collagen, procollagen, elastin, and reticulin) make up only 3–5% and are insoluble components of muscle tissue [7,9].

The alkaline or acid solubilisation and isoelectric precipitation method is widely used to obtain protein isolates. Other physical and chemical methods are also used, such as thermal extraction, homogenisation, and enzymatic hydrolysis [10]. It should be noted that there is a growing interest in more eco-friendly methods for extracting proteins from cod and seafood by-products, including ultrasound, microwave radiation, supercritical fluids, pressurised fluid extraction, pulsed electric field, steam explosion, and membrane technologies [7,11]. Perhaps these methods will soon be widely used in the food industry.

The isoelectric solubilisation/precipitation (ISP) method, also known as pH shift processing, is a widely used technique for the extraction of muscle proteins from fish (cod) by-products [12]. Muscle proteins are solubilised under extreme acidic (pH ≤ 3) or alkaline (pH ≥ 10.5) conditions. The pH of the aqueous phase, which contains the dissolved proteins, is then adjusted to the isoelectric point (pI), approximately pH 5.5 for fish muscle proteins [13]; in this case, the proteins lose solubility and precipitate. The ISP method covers the recovery of both myofibrillar and sarcoplasmic proteins, which is advantageous compared to the traditional surimi process [14]. The ISP method maintains the native protein structures [15], which prevents proteolysis and improves the functional properties, such as the solubility, emulsification, and gelling ability, of the recovered proteins [16]. Normally, sodium hydroxide is employed for alkali extraction. Replacing it with calcium hydroxide can provide a low-sodium protein that is rich in calcium [17].

To extract proteins in large volume, pH shift has been applied to by-products of cutting Atlantic cod [18], as well as mackerel [19], rainbow trout [20], silver carp [21], bighead carp [22], common carp [23] and green crab [24]. To increase the efficiency of ISP, ultrasonic vibrations are used, which ensures 94% protein recovery [14].

Zhou and Yang [25] showed that protein isolate obtained by solubilisation in an alkaline medium had better gelling properties, providing gels with high strength, compared to isolates solubilised in an acidic medium. An increase in pH (for example, from 11.5 to 12.5) decreases the gel strength of the protein [26]. Most studies have focused on the effect of pH at the alkaline dissolution stage on the functional properties of the extracted proteins, which is reflected in excellent reviews [2,27,28]. However, studies focusing on the influence of the temperature regime for alkaline dissolution on the physicochemical and functional properties of recovered fish proteins (FPIs) are practically absent.

This study aimed to determine the recovery of proteins from Atlantic cod processing waste through the isoelectric solubilisation/precipitation method (pH shift method) under different temperature and time conditions of alkaline dissolution and the effect of extraction conditions on the physicochemical and functional properties of recovered FPIs. The extraction temperature and time influence the extent of the by-reaction, namely protein hydrolysis. We hypothesised that extraction at different temperatures and for different times would affect the molecular weight distribution of the final product and, therefore, its properties. Particular attention was given to the gel-forming properties and rheological behaviour of protein isolate hydrogels as a basis for food gel production.

## 2. Results and Discussion

### 2.1. Yield and Chemical Composition of Fish Protein Isolates

Table 1 shows the yield and chemical composition of FPI-1 and FPI-2 obtained by two methods (see Section 4.2). The methods used differed in the temperature and duration of the alkaline protein solubilisation (dissolution) stage; FPI-1 and FPI-2 were extracted at low (less than 10 °C) and high (24 °C) temperatures and short (2 h) and long (20 h) processing times, respectively. In both cases, the temperatures used were below the thermal denaturation temperature of fish muscle proteins. Table 1 also shows the chemical composition of the protein-containing raw materials. The protein content of FPIs was 93.8%, and the moisture content was low—from 5.2 to 5.9%. Lipids were not detected. The product yield of FPI-1 and FPI-2 was 10.4 and 14.4%, respectively, consistent with the results obtained when extracting protein isolates from secondary protein-containing raw materials [3]. Research has been devoted to increasing the yield of the product so that the technological process is attractive for modern industry [27].

### 2.2. Functional Properties of Fish Protein Isolates

#### 2.2.1. Solubility and Zeta Potential

Water solubility is a thermodynamic and important functional characteristic of proteins. Good solubility is crucial for the production of liquid food products with a high protein content. It also helps to create conditions for the manifestation of functional properties, such as stabilisation, emulsification, foaming, and gelation [29], which are not easily achieved with insoluble proteins. The food industry uses various methods to increase the solubility of the proteins used, including protein isolates [30]. In food technology, there is typically interest in either creating completely soluble fish proteins or precipitating fish proteins under controlled conditions, such as during protein fractionation/isolation or gel formation [31]. This is also true for the method used in our work for obtaining cod protein isolates (see Section 4.2). In the first step (dissolution (solubilisation)), we create conditions for the good solubility of fish proteins, and in the second step (isoelectric precipitation), we create conditions for protein precipitation.

From the perspective of the thermodynamic foundations and colloidal perspective for protein solubility, protein–protein interactions that determine a protein’s tendency to aggregate are primarily governed by electrostatic, hydrophobic, van der Waals, hydration, and hydrogen bonding. The overall interaction potential can be described within the framework of DLVO theory [31,32]. Electrostatic and hydration interactions between protein macromolecules are repulsive and therefore promote protein solubilisation, whereas van der Waals and hydrophobic interactions are always attractive and therefore promote protein aggregation/precipitation.

It should also be noted that protein solubility is determined primarily by surface hydrophobicity and surface charge, rather than the overall hydrophobicity of the polypeptide chain. The protein surface charge can be estimated using zeta potential analysis.

pH values of the aqueous phase are a factor that has a strong influence on protein solubility. This influence is mainly due to changes in the charge (electrical characteristics) of proteins with changes in pH [33]. At pH values below or significantly above the protein’s isoelectric point (pI), strong repulsion occurs between similarly charged cationic and anionic groups along the polypeptide chains, respectively. These repulsive interactions prevent protein aggregation and determine its solubility. At its isoelectric point (strong electrostatic attraction between the cationic and anionic groups of the polypeptide chain), a sharp decrease in electrostatic repulsion causes protein aggregation due to van der Waals and hydrophobic interactions [31,32].

**Figure 1:**
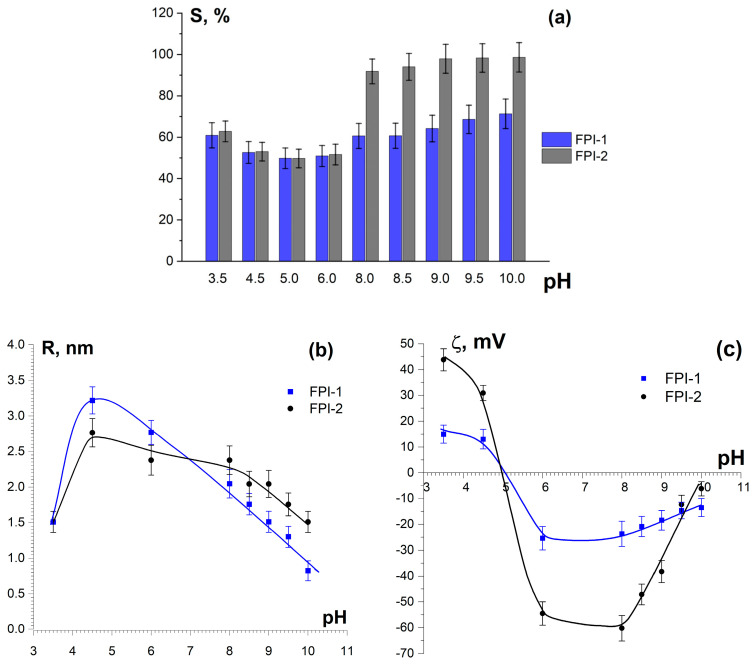
Dependence of solubility (S) (**a**), average radius (R) (**b**), and ζ-potential (**c**) of fish protein isolate particles, FPI-1 and FPI-2, on pH. C_FPI_ = 0.1%, 23 °C.

The dependence of FPI-1 and FPI-2 solubility on pH (over a wide range of 3.5–10) is shown in Figure 1a. The isolate samples demonstrate minimal solubility (<50%) in the region of the pI at pH 5.0–6.0. The pI value corresponds to pH 5.5, due to the high content of aspartic and glutamic acids in FPIs (see Section 2.3.2); these acids are characterised by pK_a_ values of approximately 3.7 and 4.1, respectively [34].

The low solubility near the pI is expected, since FPIs are obtained by pI precipitation; therefore, they contain only those protein fractions that have minimal solubility in the region of their pIs. At the same time, the zeta potential of the particles is close to zero (Figure 1c), indicating the absence of electrostatic repulsion between macromolecular chains, which induces protein aggregation due to hydrophobic and van der Waals interactions. The particle size in the pI region also shows maximum values. A sharp increase in the solubility of FPI-1 and FPI-2 was observed with a decrease in pH below the pI (at pH 3.5) and an increase in pH above the pI (at pH 8.0–10.0). At these pH values, the protein macromolecules become positively or negatively charged, the absolute value of the zeta potential increases (Figure 1c), and the particle size decreases (Figure 1b), providing strong electrostatic repulsion between the polypeptide chains and promoting the solubility of the FPI. Similar patterns have been observed, for example, for fish proteins from pollock [35] as well as plant proteins [36,37].

The solubility of FPI-2 was significantly higher (>92%) than that of FPI-1 (>61%) in a pH range of 8.0–9.5, which is suitable for food production. This may be due to the different temperature and time conditions of the alkaline dissolution of proteins during their extraction from protein-containing raw materials (see Section 4.2). The harsh temperature conditions (t = 24 °C) as well as long (20 h) processing times of extraction during the production of FPI-2 may have led to the partial destruction (hydrolysis) of the protein polypeptide chains and, accordingly, to the appearance of a high water-soluble fraction.

#### 2.2.2. Water Holding Capacity, Fat Holding Capacity, and Emulsifying Ability

The WHC and FHC are defined as the ability to retain water and oil, respectively, in an isolate sample. These properties are an important indicator of the acceptability of food quality. FPI-1, which was recovered under mild conditions at 9 °C and short (2 h) processing times (see Section 4.2), has a better ability to bind water and oil compared to FPI-2, which was recovered under harsh conditions at 24 °C and long (20 h) processing times. Thus, at pH 5.6–6.2, the WHC is 6.2 and 5.8 g/g for the first and second samples, respectively (Table 2). High WHC values of the obtained isolates indicate the possibility of their use for food product development with an increased ability to bind water and proteins [38].

The EC and EAI are determined by the volume of oil that can be emulsified by a protein before the emulsion breaks down, while the ESI characterises the resistance of emulsion droplets to coalescence. The emulsifying activity and stabilising properties of a protein depend on its solubility, hydrophobicity, molar mass, pH, ionic strength, temperature, charge, and conformational state [39].

Isolate FPI-2 showed higher emulsifying activity and emulsion stability than FPI-1. This can be explained by the high surface charge (ζ-potential) of FPI-2 particles and its high solubility at the given pH (Figure 1). The obtained values are comparable with similar characteristics of plant protein isolates [40]. Good emulsifying and stabilising properties offer the possibility of using FPIs in emulsion and moulded food products.

### 2.3. Physicochemical Properties of Fish Protein Isolates

#### 2.3.1. Molecular Weight Composition

**Figure 2:**
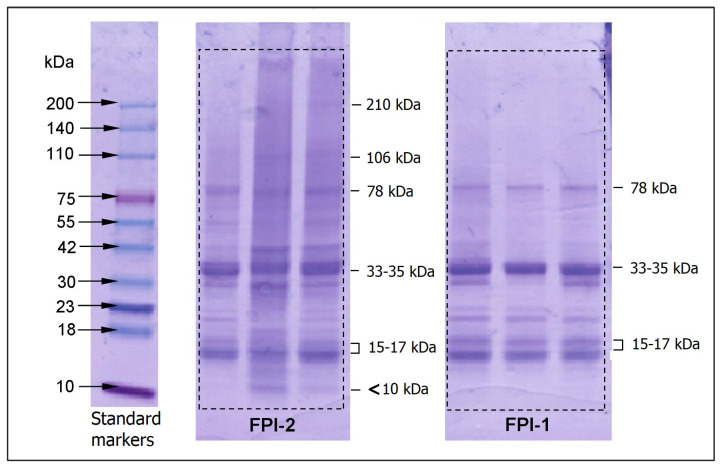
Electropherogram of protein fractions soluble in a slightly alkaline medium (at pH 8.0).

The protein profiles (molecular weight composition) of soluble fractions of FPI-1 and FPI-2 were analysed using SDS-PAGE (Figure 2). Before the analysis, isolates were dissolved in water at pH 8.0.

Comparison of the FPI-1 and FPI-2 profiles showed that there were no significant qualitative differences between the soluble fractions of the obtained isolates. The final profile mainly contained bands attributed to the myosin light chain (15–17 kDa) and myoglobin (17 kDa). A band in the 30 kDa region and an intensely coloured wide band in the 33–35 kDa region was attributed to tropomyosin (30–36 kDa). There were bands attributed to troponin T (41 kDa) and actin (43 kDa). The protein profile of FPI-2 contained a band for the low-molecular protein parvalbumin (6 kDa) [41]. In addition, FPI-2 electropherograms contained intense bands in the range of 78–80 kDa, corresponding to myogen (81 kDa).

It is also necessary to note the presence of diffuse bands of low intensity, corresponding to proteins with a higher molecular weight, namely actinin (106 kDa) and heavy chains of myosin (210 kDa) [18]. These high molecular weight protein bands are barely visible in the profile of FPI-1 recovered at low temperature. Considering the low solubility of FPI-1 at pH 8 (see Section 2.2.1), it can be assumed that the obtained protein profile of this isolate (Figure 2) may not accurately reflect the presence of all protein fractions, considering the undissolved part. Apparently, dissolving the FPI-1 sample in a slightly alkaline solution (pH 8) is insufficient to completely solubilise the myosin heavy chain. As a result, heavy chains of myosin and other myosin-associated bands are not visible in the gel. In future studies, we plan to dissolve the sample in a solution containing SDS to ensure the participation of virtually all proteins in SDS-PAGE.

The obtained result is similar to the results previously published for Antarctic krill [42]. A study of protein isolates extracted from common carp showed that the increased zeta potential, emulsion activity, and foam stability observed in alkaline protein isolates correlated with increased levels of 146, 40, and 37 kDa polypeptides [43].

Myosin, tropomyosin, troponin T, and actin are myofibrillar proteins and are important in muscle tissue, including fish; myosin makes up approximately 60% of muscle protein. The myofibrillar proteins, primarily myosin, largely determine the ability of the isolated protein isolate to from a gel [44]. The observed higher content of low molecular weight protein fractions in FPI-2 is in good agreement with its high solubility (see Section 2.2.1).

#### 2.3.2. Amino Acid Composition

The nutritional and biological value of protein is determined by the type and quantity of amino acids, primarily essential amino acids. The amino acid profile of FPIs obtained by two methods from Atlantic cod processing waste, as well as the amino acid composition of the raw material (cod muscle tissue), is presented in Table 3. The amino acid composition of FPI-1 and FPI-2 was almost identical and corresponds to the amino acid profile of the original raw material. A high content of aspartic and glutamic acids is shown, which is typical for FPIs [4]. Thus, the proportion of glutamic acid was 17 and 16% for FPI-1 and FPI-2, respectively. A high glutamic acid content is also noted in protein isolates from anchovy [45] and arctic krill [42].

Both fish isolates, FPI-1 and FPI-2, were characterised by a high content of essential amino acids (41.9 and 41.8 g/100 g protein, respectively), which met the requirement of the Food and Agriculture Organization of the United Nations/World Health Organization/United Nations University (FAO/WHO/UNU) [1] for both adults and children [46]. The isolates showed high levels of essential acids, such as leucine, lysine, threonine, valine, and isoleucine; of these, leucine is present in the largest amount at 14.4 g/100 g protein. Methionine is the limiting amino acid in FPI-1 and FPI-2, as the content of this amino acid is below the FAO/WHO/UNU standards (Table 3). Essential acids regulate nutrient metabolism and support the human body’s immunity [4]; hydrophobic amino acids can act as antioxidants [47].

**Table 3:** Amino acid compositions of fish protein isolate (FPI-1 and FPI-2) from Atlantic cod (content of amino acid in g/100 g protein).

Amino Acids	Cod Muscle Tissue [48]	FPI-1	FPI-2	FAO/WHO/UNU Standards for Adults (g/100 g Protein) [46]
Glycine	4.7 ± 0.1 ^a^	3.7 ± 0.1 ^b^	3.6 ± 0.3 ^b^	
Proline	3.7 ± 0.3 ^a^	2.3 ± 0.5 ^b^	2.4 ± 0.6 **^b^	
Aspartic acid	7.9 ± 1.3 ^a^	9.1 ± 0.2 ^b^	9.2 ± 1.1 ^b^	
Glutamic acid	17.8 ± 1.5 ^a^	17.6 ± 0.6 ^a^	15.5 ± 1.5 ^b^	
Serine	4.9 ± 0.2 ^a^	4.0 ± 0.2 ^b^	3.5 ± 0.4 ^c^	
Histidine	2.0 ± 0.1 ^a^	5.3 ± 0.2 ^b^	6.3 ± 0.4 ^c^	1.5
Threonine *	4.7 ± 0.1 ^a^	4.1 ± 0.1 ^b^	3.5 ± 0.4 ^c^	2.3
Arginine	7.2 ± 0.4 ^a^	7.3 ± 0.2 ^a^	8.4 ± 0.3 ^b^	
Alanine	6.4 ± 0.3 ^a^	5.2 ± 0.1 ^b^	5.3 ± 0.4 ^b^	
Tyrosine	4.0 ± 0.2 ^a^	6.1 ± 0.3 ^b^	7.0 ± 0.5 ^c^	3.8
Valine *	4.8 ± 0.2 ^a^	6.6 ± 0.1 ^b^	7.2 ± 0.4 ^c^	3.9
Methionine *	3.0 ± 0.2 ^a^	1.5 ± 0.2 ^b^	1.5 ± 0.1 ^b^	2.2
Leucine *	8.9 ± 0.3 ^a^	14.4 ± 0.4 ^b^	14.4 ± 0.5 ^b^	5.9
Isoleucine *	4.2 ± 0.2 ^a^	3.9 ± 0.1 ^a^	4.0 ± 0.2 ^a^	3.0
Lysine *	10.2 ± 0.5 ^a^	8.0 ± 2.6 ^b^	7.8 ± 2.7 **^b^	4.5
Phenylalanine *	4.2 ± 0.2 ^a^	3.4 ± 0.1^b^	3.4 ± 0.2 ^b^	3.8
∑ * (EAA)	40.0 ± 0.7	41.9 ± 2.6 ^a^	41.8 ± 2.7	

*—essential amino acids (EAA). **—determined by capillary electrophoresis in FBI “Test-St. Petersburg”. Superscript letters indicate significant differences (*p* < 0.05) among the groups. Means ± standard deviation (*n* = 3).

The obtained results allow us to consider FPI-1 and FPI-2 from cod as a protein ingredient with high nutritional value in the creation of food products.

### 2.4. Characteristics of Heat-Induced Hydrogels from Fish Protein Isolate

#### 2.4.1. Thermal Analysis

**Figure 3:**
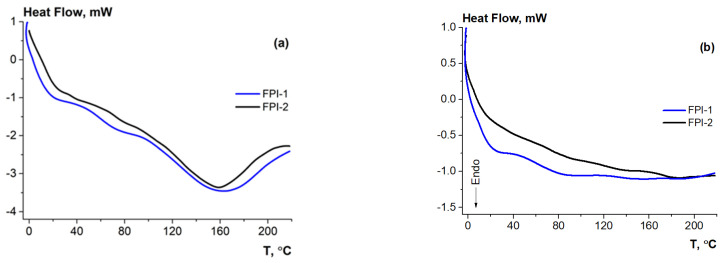
Thermograms of the first (**a**) and second (**b**) heating cycles of fish protein isolates.

The DSC method was used to study the thermal properties of the obtained FPIs, including glass transition and protein denaturation upon heating. Figure 3 shows the DSC thermograms for two heating cycles of FPI-1 and FPI-2 from 0 to 220 °C at a heating rate of 20 °C/min.

A stepwise change in the heat flux in a certain temperature range of the first heating cycle (Figure 3a) was used to determine the glass transition temperature (T_g_) of the biopolymer. Glass transition is considered as the transition of an amorphous material from a glassy (solid-like) to a rubbery state. Being a typical glass → elastomer relaxation transition for amorphous polymers, glass transition leads to a change in the number of degrees of freedom during conformational changes in the macromolecular coil, which is accompanied by a change in the heat capacity of the system. The T_g_ was determined in the middle of the temperature region in which a stepwise change in the heat flux occurred on the DSC curve. The T_g_ of FPI-1 and FPI-2 are given in Table 4 and are 70.26 and 64.55 °C, respectively. Similar results were demonstrated for protein isolates from defatted Antarctic krill [42], where it was shown that T_g_ was 50 °C at a heating rate of 50 °C/min. The dependence of the glass transition temperature on the heating rate was noted.

At temperatures exceeding T_g_, endothermic peaks corresponding to heat-induced denaturation of myofibrillar proteins of fish isolates were observed in the DSC curves of the first heating cycle (Figure 3a). Denaturation is associated with structural changes in protein macromolecules and a conformational transition from an ordered to a disordered state of polypeptide chains. Such changes are necessary, for example, for further reorganisation of protein molecules of the isolate during gel formation under subsequent cooling conditions [49]. Therefore, quantifying the thermal transition of FPIs is useful in discussing their gelation ability.

The initial peak temperature (T_b_), denaturation temperature (T_d_), and thermal transition enthalpies (ΔH_g_) are given in Table 4. Comparison of the obtained values showed higher Td values for FPI-1 than for FPI-2. Thus, Td was 163.0 and 158.5 °C (heating rate 20 °C/min), respectively. The obtained results may be due to the mild conditions of alkaline extraction of FPI-1 for forming biopolymers with a high molecular weight (see Section 2.3.1) and a more ordered conformation, which increases the thermal stability of the isolates [44]. FPI-1 demonstrates a higher enthalpy of denaturation (Table 1), which also confirms higher resistance to denaturation [50] compared to FPI-2, which was obtained at a high temperature. The effect of the heating rate on Td values is noted, which is similar to the result obtained by [42].

The DSC thermograms of the second heating cycle (Figure 3b) demonstrated the absence of any peaks, indicating the irreversibility of conformational transformations during heat-induced protein denaturation.

#### 2.4.2. Protein Secondary Structure from FT-IR Spectra

**Figure 4:**
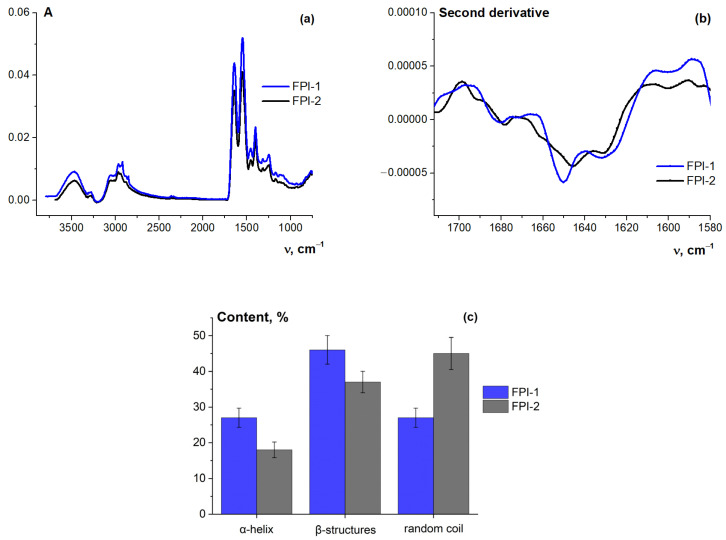
(**a**) FT-IR spectra of FPI-1 and FPI-2 solutions, (**b**) the second derivative of the absorption spectra in the Amid I region, and (**c**) content of the Amid I components (secondary structures) for fish protein isolates. C_FPI-1_ = C_FPI-2_ = 5%, T = 25 °C.

Figure 4a shows the FT-IR spectra of FPI-1 and FPI-2. The spectra contain all the characteristic absorption bands inherent in proteins [51] and FPIs [18]—Amid A (3400–3300 cm^−1^) corresponding to stretching vibrations of N–H and O–H groups and Amid B (3000–2900 cm^−1^) corresponding to the stretching vibrations of N–H bond atoms; Amid I (1710–1580 cm^−1^) corresponding to stretching vibrations of C=O and C–N bonds; Amid II (1575–1480 cm^−1^) corresponding to deformation vibrations of N–H groups and stretching vibrations of C–N groups; and Amid III (1300–1230 cm^−1^) corresponding to stretching vibrations of N–H and C–N groups.

FT-IR spectroscopy was used to evaluate the secondary structure of the FPIs. The shape and position of the Amid I peak are sensitive to the secondary structure of proteins [52]. The complex contour of Amid I was described by the sum of the individual components, the number and position of the maximum determined using the second derivative (Figure 4b). According to Kong and Yu [53], the following assignment of bands was made to identify different secondary structures (components) of the protein: 1654 cm^−1^—α-helices; 1619, 1631, and 1688 cm^−1^—β-sheets; 1665 and 1676 cm^−1^—β-turns; and 1643 cm^−1^—disordered structure (random coil).

For quantitative determination of different structures, the ratio of the integral intensity (area) of an individual contour describing the corresponding structure to the sum of the individual component areas was used. The results of the decomposition are shown in Figure 4c. Of greatest interest is the content of helices and random coil; the content of β-structures is the sum of the content of β-turns and β-sheets. In this study, a significance level of *p* < 0.050 was adopted to define statistical significance.

Analysis of the protein secondary structure in FPI-1 of a higher molecular weight and FPI-2 of a lower molecular weight shows that the first sample is characterised by a high ordered α-helix content and low disordered random coil structure content compared to the second sample. Thus, the α-helix is 27 and 18%, and the random coil is 27 and 45% for FPI-1 and FPI-2, respectively. A similar result was obtained in the study of protein isolates from tilapia by IR spectroscopy and Raman spectroscopy [54]. Both methods showed that the degree of protein structure unfolding increased when isolates were prepared at 25 °C, compared to samples prepared at 5 °C.

The obtained data (Figure 4c) are in good agreement with the DSC results (see Section 2.4.1), characterising the thermal stability of the isolates. The secondary structure of the native protein as well as the molecular weight distribution is responsible for the manifestation of functional properties. Thus, the presence of helical sections of the macromolecular chain is mainly responsible for the protein’s ability to form gel.

#### 2.4.3. Rheological Behaviour

**Figure 5:**
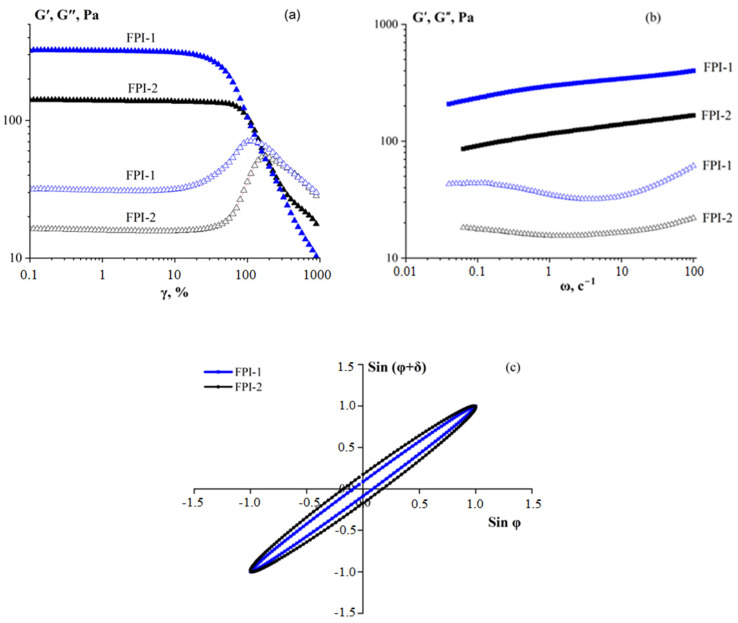
Amplitude sweep (**a**), frequency sweep (**b**), and Lissajouse–Bowditch figures (**c**) of hydrogels made of fish protein isolates FPI-1 and FPI-2; open symbols—storage modulus; full symbols—loss modulus. C_FPI-1_ = C_FPI-2_ = 5%, t = 4 °C.

Rheological properties are an important characteristic of food gels based on FPIs. The characteristics of the viscoelastic properties of isolate hydrogels (C_FPI-1_ = C_FPI-2_ = 5%) obtained in dynamic mechanical analysis mode are presented in Figure 5. The strain amplitude sweeps (Figure 5a), as a large-amplitude oscillatory shear (LAOS) test [55], show that the storage modulus (G′) exceeds the loss modulus (G″) and that elastic behaviour dominates over the viscous behaviour in the linear viscoelastic region. The FPI-1 and FPI-2 hydrogels demonstrated linear viscoelastic behaviour over a wide range of strain amplitudes up to approximately 65 and 20%, respectively.

The completion of the linear response for gels with increasing deformation amplitude is determined by a decrease in the storage modulus. At a certain critical deformation (γ*) G′ = G″, which corresponds to the transition of samples between a solid-like and liquid state and allows us to classify the studied systems as yielding media [56]. A similar response to the deformation sweep has been observed in various biopolymer hydrogels [28,57], including FPIs [18,58]. The data obtained showed an insignificant decrease in γ* for the FPI-1 hydrogel compared to FPI-2, indicating a decrease in stability under vibration deformation as a result of structural features in the FPI-1 gels.

The results for the frequency sweep tests of the FPI hydrogels, plotting the storage modulus and loss modulus on the frequency in the linear region of the viscoelastic behaviour are shown in Figure 5b. The storage modulus exceeded the loss modulus G′ > G″, which demonstrated the solid-like behaviour of the systems [59]. Similar results were obtained for protein isolates from carp [43]; it was confirmed that the rheological properties of protein isolates from male carp were superior to those from females, resulting in superior functional properties.

The rigidity (elastic modulus) of the FPI-1 gel was more than twice as high as the rigidity of the FPI-2 gel. This result is in good agreement with the data obtained by electrophoresis, IR spectroscopy, and DSC. The effect of a higher content of high molecular fractions, ordered conformation of polypeptide chains, and increased thermal stability in FPI-1 compared to FPI-2 leads to a compact and strengthened three-dimensional hydrogel network.

The viscoelastic properties of the material can also be characterised by Lissajouse–Bowditch figures [60]. Two examples of such figures, created for the FPI-1 and FPI-2 gels (C_FPI-1_ = C_FPI-2_ = 5%) at a deformation of 1% and different frequencies, are shown in Figure 5c. The Lissajouse–Bowditch figures are ellipses. Analysis of the obtained figures showed that the area of the ellipse for the FPI-1 gel was much smaller than the area for FPI-2. This means that the response of the materials was viscoelastic, while the response of the FPI-1 gel was predominantly elastic. The increase in the figure area for FPI-2 reflects the growth of dissipative losses.

We compared our results with the previously obtained rheological properties of gelatine-based hydrogels, including mammalian gelatines from bovine and porcine skin, and fish gelatines from commercial samples and samples extracted from Atlantic cod skin in our laboratory [61]. Mammalian gelatines provide high strength and elasticity to the hydrogels. Fish gelatine from cod skin also provides high strength to the hydrogels. It is worth noting that the strength and elasticity (elastic moduli, Figure 5a) of hydrogels made from high-molecular-weight FPI-1 are comparable to those of gels made from mammalian gelatines.

#### 2.4.4. Microstructure Analysis

**Figure 6:**
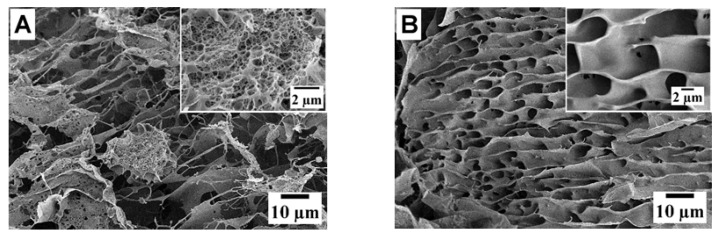

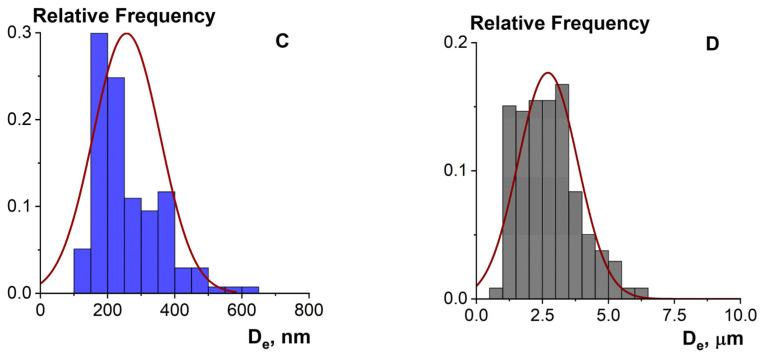
SEM images of gels (xerogels) of fish protein isolate FPI-1 (**A**) and FPI-2 (**B**); diagrams of cell size distribution in gels of isolates FPI-1 (**C**) and FPI-2 (**D**).

To confirm the formation of a denser structural network for FPI-1 gels compared to FPI-2 gels, we used the SEM method. The SEM visualisation of the morphology of lyophilised hydrogels of FPIs at different scales is presented in Figure 6A–D.

As shown in the obtained SEM images, both isolates formed a three-dimensional network structure with noticeable differences in morphology and pore sizes. The high-molecular fish isolate FPI-1 formed an irregular dense structure with frequent interchain contacts and small cell sizes of about 250 nm (Figure 6C). The low-molecular fish isolate FPI-2 demonstrated a more regular honeycomb structure, with clearly defined pores of large sizes. The average pore size was about 3 μm (Figure 6D). The structure was characterised by a uniform distribution of pores on a micrometre scale and a less frequent network of intermolecular contacts.

Comparative analysis of the structure of FPI-1 gels and, for example, porcine gelatine [61] reveals a similar dense gel structure, formed by thick tightly packed fibres and characterised by low porosity.

## 3. Conclusions

Two samples of FPI were extracted from Atlantic cod by-products (muscle tissue) by the isoelectric solubilisation/precipitation method under mild conditions of alkaline dissolution at temperatures not exceeding 10 °C for 2 h and under harsh conditions at temperatures of 24 °C for 20 h, resulting in FPI-1 and FPI-2, respectively. The feasibility of recovering fish proteins from cod by-products for use as food ingredients has been demonstrated. The temperature regime and processing time affected the molecular weight distribution of the extracted proteins. FPI-1, recovered under mild conditions, was characterised by a high content of high-molecular fractions; in contrast, isolate FPI-2, recovered under harsh conditions, contained a large number of low-molecular fractions. High solubility in the water of low-molecular FPI-2 at pH 8–9 (more than 92%) was noted in comparison with high-molecular FPI-1 (about 60%), which only dissolved at pH 10 and above. The low-molecular sample exhibited high emulsifying properties.

The HPLC method showed that the amino acid profile of FPIs was similar to the profile of protein-containing raw material, namely cod muscle tissue, and was characterised by the presence of all essential amino acids, including lysine, leucine, and isoleucine, as well as glutamic and aspartic acids. Analysis of the thermal properties (according to differential scanning calorimetry) showed high denaturation temperatures of high-molecular FPI-1 compared to low-molecular FPI-2, with values of 163.0 and 158.5 °C, respectively. The increased thermal stability of FPI-2 was due to a more ordered conformation of the protein recovered under mild temperature conditions. This was confirmed by IR spectroscopy data in the analysis of the secondary structure of protein isolates. FPI-1 was characterised by a high α-helix content and a low random coil content compared to FPI-2 (27 versus 18% and 27 versus 45%, respectively).

The elastic modulus and strength of the FPI-1 gel was more than twice that of the FPI-2 gel. The effect of a higher content of high-molecular fractions, ordered conformation of polypeptide chains, and increased thermal stability of FPI-1 compared to FPI-2 led to the compaction and strengthening of the hydrogel network. SEM micrographs recorded a dense three-dimensional structure with frequent interchain contacts and small cell sizes of about 250 nm for the high-molecular FPI-1 gel. The low-molecular FPI-2 gel demonstrated a more regular structure with large pores of about 3 μm. The obtained FPIs can be used as food ingredients or food additives in the production of a wide range of food products (sausages, pâtés, puddings, mousses, sauces, etc.) due to their excellent nutritional and functional properties (solubility, emulsifying, foaming, binding, and gelling abilities) as well as gelling properties. Future studies are planned to examine the effect of IBP on the organoleptic properties, shelf stability, and other technological parameters of food products produced using IBP. These studies will expand our understanding of the potential uses of fish protein isolates and their impact on various product characteristics.

## 4. Materials and Methods

### 4.1. Materials

Waste from the industrial processing of Atlantic cod (*Gadus morhua*) was used as the protein-containing raw material for obtaining FPIs.

Atlantic cod was caught by Trawl Fleet Co., Ltd. (Murmansk, Russia) in the Central–Eastern region of the Atlantic, frozen, and delivered to the port of Murmansk to an industrial enterprise, where the fish were processed to obtain semi-finished fish products, including skinned fish fillets. The waste was meat cuttings, i.e., pieces of fish muscle tissue of arbitrary shape and size without skin or with skin without scales. We received the waste from the industrial enterprise; then, we crushed the waste (to obtain fish mince), froze it, and stored it at a temperature not exceeding −18 °C; the storage time was no more than 6 months.

### 4.2. Preparation of Fish Protein Isolate

**Figure 7:**
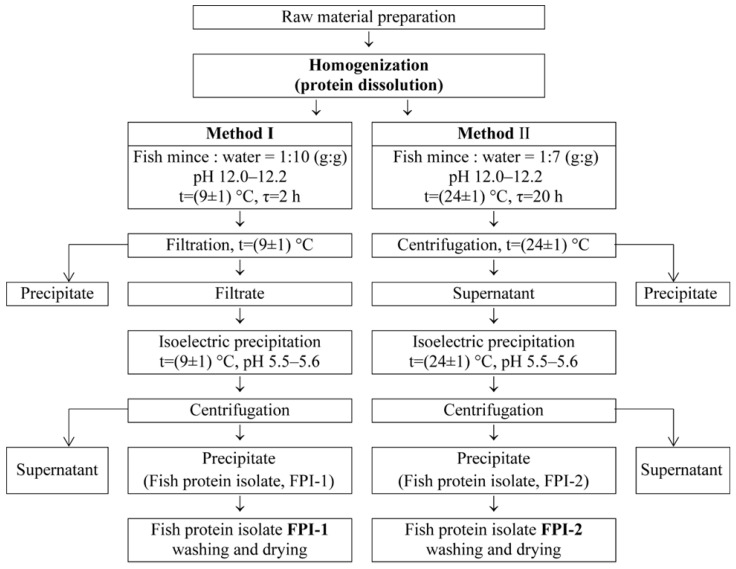
A flowchart for the recovery of two fish protein isolates (FPI-1 and FPI-2) using the isoelectric precipitation method.

FPIs were obtained using the isoelectric precipitation method [62] with some modifications concerning the stage of obtaining the water-soluble protein fraction from minced fish. A flowchart for FPI recovery is shown in Figure 7. Two methods were used to carry out the homogenisation stage (protein dissolution), and two FPI samples, FPI-1 and FPI-2, were recovered.

The main stages of FPI extraction were carried out as follows.

*Raw material preparation*. Cod mince was defrosted and washed twice with cold water. For this purpose, the mince was mixed with water in a mass ratio of mince:water = 1:2, stirred for 5 min, and filtered through 4 layers of gauze fabric.

*Protein dissolution (solubilisation)*. Water-soluble protein was extracted from the washed mince by alkaline dissolution using two methods. In method 1, the washed mince was mixed with distilled water in a weight ratio of 1:10, and the pH of the mixture was adjusted to 12.0–12.2. The mixture was homogenised for 2 h with constant stirring at a temperature of no more than 10 °C. The insoluble protein fractions were separated using filtration through 6 layers of gauze fabric. The filtrate was sent for isoelectric precipitation. Upon further processing, the FRI-1 sample was obtained.

In method 2, the washed mince was mixed with distilled water in a weight ratio of 1:7, and the pH of the mixture was adjusted to 12.0–12.2. The mixture was homogenised for 4 h with constant stirring at a temperature of 24 ± 1 °C. The mixture was kept at this temperature for 20 h without stirring. The insoluble protein fractions were separated using centrifugation (centrifuge UC-1536E ULAB (Xieli International Trading Co., Ltd., Hangzhou, Zhejiang, China), speed—5000 rpm, time—40 min). The clarified protein solution (supernatant) was sent for isoelectric precipitation. Upon further processing, the FRI-2 sample was obtained.

In both the first and second methods, the temperatures used were below the thermal denaturation temperature of fish proteins [31].

*Isoelectric precipitation*. The pI of most muscle proteins is approximately pH 5.5, at which point the electrostatic repulsion between protein macromolecules is minimal. At this pI, protein–protein bonds are strengthened, and protein–water interactions are hindered, leading to protein precipitation. Protein precipitation was carried out at room temperature and constant stirring. The pH of the reaction medium was adjusted to pH 5.5–5.6 using 2 M HCl. The system with the precipitated proteins was then dehydrated by centrifugation (centrifuge UC-1536E ULAB, speed—3000 rpm, time—4 min).

The resulting sediment, which was FPI, was washed twice with distilled water and dried in a freeze dryer BK-FD10T (Biobase, Jinan, China) at −50 °C. The resulting product was stored at 5 ± 1 °C. The yield of FPI (B, %) was calculated using the following equation:

(1) $$B=\frac{m_{FPI}}{{\;\;\;m}_{F}}\times 100\%,$$

where m_F_ (g) and m_FPI_ (g) are the mass of the wet raw materials and dried FPI, respectively.

### 4.3. Physicochemical Properties of Fish Protein Isolates

#### 4.3.1. Chemical Composition

By analysing the raw materials and FPI samples, we determined the total nitrogen, lipid, moisture, and mineral content using standard methods [63]. The total nitrogen was determined by the Kjeldahl method, lipid—by the Soxhlet method, moisture—by the gravimetric method, and mineral substances—by the combustion method.

#### 4.3.2. Amino Acid Profile by High-Performance Liquid Chromatography (HPLC)

High-performance liquid chromatography was used to obtain the amino acid profile of the isolates. A Biochrom 30+ chromatograph (Biochrom Ltd., Cambridge, UK) was used, along with a 4.6 × 150 mm column. A ninhydrin solution (Sevko&Co, Moscow, Russia) served as the derivatising agent; the feed rate was 20 mL/h. The reaction cell temperature was 135 °C. The injected sample volume was 20 μL. Detection was performed at wavelengths of 440 and 570 nm. The system was calibrated in the concentration range of 0–100 mg/L using standard solutions prepared from individual amino acids (Sigma-Aldrich, St. Louis, MO, USA) by the gravimetric method. A sodium solution for sample dilution (Sevko&Co, Moscow, Russia) was used as a solvent.

#### 4.3.3. Molecular Weight Composition by Vertical Electrophoresis (SDS-PAGE)

The molecular weight composition of protein fractions soluble in a slightly alkaline medium (at pH 8.0) was estimated by vertical electrophoresis in polyacrylamide gel using the surfactant—sodium dodecyl sulphate (SDS). The following buffer solutions were used for the analysis: running buffer (pH 8.3) and sample buffer (pH 6.8). The running buffer consisted of 25 mM Tris, 192 mM glycine, and 1% SDS. The running buffer was diluted 10-fold before use. The sample buffer consisted of 62.5 mM TriS-HCl, 2% SDS, 25% glycerol, 0.01% bromophenol blue, and 5% β-mercaptoethanol. Mini-PROTEAN TGX Gels Plus gradient polyacrylamide plates with a gel concentration of 4–20% (BIO-RAD, Berkeley, CA, USA) were used to separate the proteins. FPI suspensions were prepared with a concentration of 1 wt.% and pH 7.9–8.0. The suspension was thoroughly mixed at room temperature for 120 min and kept at a temperature of 5 ± 1 °C for 14 h. The undissolved isolate was removed by centrifugation (at 5000 rpm for 10 min). The resulting solution (supernatant) was mixed with a buffer solution, maintained at 95 °C for 5 min, and put on a polyacrylamide gel.

Separation of protein fractions was carried out with a current strength of 20 mA and voltage of 100 V. The gel plate was then treated with an acetic–alcohol solution for 30 min, and the proteins were stained using the Coomassie method. To determine the molecular masses of protein fractions, electrophoretic mobility analysis of standard proteins (Servicebio Technology, Wuhan, Hubei Province, China) with molecular masses ranging from 10 to 200 kDa was used.

#### 4.3.4. Zeta Potential and Particle Size by Dynamic Light Scattering

The zeta potential and particle size of FPI solutions were determined by electrophoretic and dynamic light scattering using Photocor Complex ZI (Photocor, Moscow, Russia). The light source was a thermally stabilised semiconductor laser (λ = 636.8 nm, 35 mW). The zeta potential and hydrodynamic radius of particles were determined at light scattering angles of 20° and 90°. The accumulation time of the Doppler signal phase function was 5 min. For the experiment, aqueous (pH 8.0) solutions of FPI-1 and FPI-2 were prepared with a concentration of 0.05 wt.%; the solutions were filtered using CHROMAFIL Xtra PET syringe polyamide filters (Macherey-Nagel, Düren, Germany) with a membrane pore size of 0.45 μm. The analysis of samples was performed in triplicate.

### 4.4. Functional Properties of Fish Protein Isolates

#### 4.4.1. Water Holding Capacity and Fat Binding Capacity

The water holding capacity (WHC) and fat holding capacity (FHC) of FPIs were determined using the gravimetric method [64]. For the WHC, the FPI and distilled water (or phosphate buffer solution with pH 7.2) were placed in dry centrifuge tubes with a mass ratio of 1:100. The contents of the tubes were mixed using a shaker at 150 rpm for 2 h and held at room temperature (20–25 °C) for 6–8 h for swelling. Excess solvent was removed by centrifugation (at 3000 rpm for 10 min), and the contents of the tubes were weighed. A similar procedure was used to determine FHC; only, instead of water, premium vegetable oil was placed in the dry centrifuge tube along with FPI. Subsequently, the WHC (g/g) and FHC (g/g) were calculated based on following equations:

(2) $$WHC=\frac{m_{1}}{m_{2}},$$

(3) $$FHC=\frac{m_{1}}{m_{2}},$$

where m_1_ (g) is the mass of the FPI sample after swelling and centrifugation, and m_2_ (g) is the mass of the initial dry FPI sample.

#### 4.4.2. Solubility

The solubility of FPI in water was determined at different pH values. Suspensions of 1 wt.% FPIs were prepared, and the pH was adjusted to the required values using 2 M and 0.1 M NaOH. The suspensions were thoroughly mixed at room temperature for 120 min and kept at 5 ± 1 °C for 12–14 h. The undissolved isolate was removed by centrifugation (speed—5000 rpm, time—10 min). The supernatant was dried, and the total nitrogen content was determined in the dried supernatant using the Kjeldahl method. The solubility (S, %) of the FPIs was calculated using the following equation:

(4) $$S=\frac{N_{T_{1}}}{N_{T_{2}}}\;\times \;100\%,$$

where N_T1_ (wt.%) and N_T2_ (wt.%) are the total nitrogen content in the dried supernatant and in the FPI, respectively [65].

#### 4.4.3. Emulsifying Capacity

The emulsifying capacity (EC) was determined using the method proposed by [66] with minor modifications. Suspensions of 1 wt.% FPI with pH 8.0 were prepared (see Section 4.4.2), and the undissolved isolate was removed by centrifugation. The clarified protein solution was used to prepare the emulsions. The emulsions were obtained by dispersing the aqueous and oil phases (volume ratio 50:50) using a T25 Digital Ultra-Turrax IKA disperser (IKA, Staufen, Germany) at a stirring speed of 8000 rpm for 10 min at room temperature (~23 °C). Refined and deodorised premium sunflower oil was used as the oil phase. A fixed volume of the emulsion (40 mL) was placed in centrifuge tubes and centrifuged at 4000 rpm for 5 min. The EC (%) of the FPIs was calculated with the following equation:

(5) $$EC=\frac{V_{CE}}{V}\;\times \;100\%,$$

where V_CE_ (cm^3^) is the volume of concentrated emulsion after centrifugation, and V (cm^3^) is the volume of emulsion before centrifugation.

The emulsion activity index (EAI) and the emulsion stability index (ESI) were calculated using the method proposed by [40]. Isolate solutions with C_FPI_ = 1% wt. and pH = 8.0 were prepared, as described in Section 4.4.2. To obtain the emulsion, the FPI solution was mixed with premium sunflower oil in a volume ratio of 3:1 using a T25 Digital Ultra-Turrax disperser at 20,000 rpm/min. At different times (immediately after preparation and after 10 min), 50 μL of the resulting emulsion was collected and mixed with 5 mL of a 0.1% (*w*/*v*) sodium dodecyl sulphate solution. The absorbance of the diluted emulsion was then measured at 500 nm using a Unico 2800 UV VIS Spectrophotometer (United Products & Instruments, Dayton, NJ, USA). The EAI (m^2^/g) and ESI (min) were calculated as follows:

(6) $$AI\left(m^{2}/g\right)=\frac{2\;\times \;2.303\;\times \;A_{0}\cdot B}{C\;\times \;\varphi \;\times \;L},$$

where B is the emulsion dilution factor (B = 100); C (g/m^3^) is the isolate concentration; φ is the concentration of the initial emulsion (oil concentration in the emulsion, φ = 0.25); L is the optical path length (L = 0.01 m); and A_0_ is the absorption (optical density) of the emulsion measured immediately after preparation.

(7) $$ESI\left(min\right)=\frac{A_{10}\;\times \;\Delta t}{A_{0}\;-\;A_{10}},$$

where A_0_ and A_10_ are the absorption capacity of the emulsion after 0 and 10 min, respectively (Δt = 10).

### 4.5. Preparation of Heat-Set Fish Protein Isolate Gels

Aqueous dispersions of 5 wt.% FPI with a pH of 7.2–8.0 were prepared. The dispersions were thoroughly mixed, placed in sealed containers (bowls with ground-glass lids), and kept at a temperature of 4–5 °C for 20 h. The samples were then degassed. The bowls with FPI samples were heated in a water bath at 93–94 °C for 20 min [67]. Following heating, the bowls were cooled to 0 °C, and the FPI gels were kept at 4–5 °C for 20 h before analysis.

The hydrogel formed from FPI-1 is translucent in appearance and light yellow in colour; the hydrogel formed from FPI-2 is more transparent and lighter in colour with a yellowish tint. The gels have a uniform structure, without visible inclusions or granularity. The gels are dense but not hard; the consistency is jelly-like. When mechanically pressed, they yield but retain their shape.

### 4.6. Physicochemical Properties of Heat-Set Fish Protein Isolate Gels

#### 4.6.1. Thermal Properties by Different Scanning Calorimetry (DSC)

Thermal properties of films of lyophilised FPIs were studied by differential scanning calorimetry (DSC) using a DZ-DSC300C differential scanning calorimeter (NanJing Dazhan Testing Instrument, Nanjing, China) with sealed aluminium crucibles. The instrument was calibrated by measuring the heat and melting point of pure indium. To obtain the DSC curve, 4–6 mg of sample was placed in the crucible and hermetically sealed. Preliminary research showed that no differences in thermograms were observed when the sample weight changed from 4 to 6 mg. An empty crucible was used as a reference sample. The heat flux used was in the range of 50–250 °C, and the heating rate was 10 °C/min. The following measurement protocol was also used: heating rate of 20 °C/min; (1) cooling to 0 °C, (2) holding at 0 °C for 5 min, (3) first heating cycle: heating from 0 to 220 °C, (4) cooling from 220 to 0 °C, (5) second heating cycle: heating from 0 to 220 °C. The experiment was carried out in a nitrogen atmosphere with a gas flow rate of 25 mL/min. All DSC measurements were performed in triplicate.

#### 4.6.2. Protein Secondary Structure by FT-IR Spectroscopy

The infrared (IR) absorption spectra were recorded on a Fourier transform infrared (FT-IR) spectrophotometer (model Invenio, Bruker Corporation, Billerica, MA, USA) in the frequency range from 4000 to 600 cm^−1^, at a spectral resolution of 4 cm^−1^, with a thermostatically controlled attenuated total reflection attachment with a ZnSe crystal. The studied samples were thermostated at 25 °C. The FPI solution (C = 5%, V = 20 μL) was applied to the surface of the ATR measuring element, and the spectrum was recorded. The IR spectrum of the Amid I band (1580–1710 cm^−1^) was treated using Fityk 1.3.1 [68]. The Amid I band was decomposed into several components (secondary structures of the protein: helices, β-sheets, β-turns, and random coil) using a Gaussian distribution [69,70].

#### 4.6.3. Rheological Characteristics

The rheological characteristics of the hydrogels were measured using a Physica MCR 302 rheometer (Anton Paar GmbH, Graz, Austria) with a cone-and-plate measuring cell. Detailed steps followed Malkin and Isayev [60]. A strain amplitude sweep (γ was varied from 10^−1^ to 10^3^%) at constant frequency f = 1 Hz was conducted. Values of the storage modulus Gʹ were recorded to find the region of the linaer viscoelastic behaviour for all samples. A frequency sweep (ω was varied from 5 × 10^−2^ to 10^2^ rad/s) at a constant strain of 1% was conducted. Changes in the storage modulus G′ and loss modulus G″ values were measured.

All measurements were performed at 4.00 ± 0.03 °C. The sample temperature was controlled using Peltier elements P-PTD200/GL (Anton Paar GmbH, Graz, Austria).

#### 4.6.4. Microstructure by Scanning Electron Microscopy (SEM)

The morphology of lyophilised hydrogels of FPIs was studied by scanning electron microscopy (SEM) using a Merlin field emission scanning electron microscope (Carl Zeiss, Oberkochen, Germany). The experiments were carried out at an accelerating voltage of 5 kV using cryogels from the prepared FPIs. The prepared hydrogels (see Section 4.5) were left overnight at 4–6 °C. The samples were then frozen in liquid nitrogen and vacuum freeze-dried in a Labfreez FD-10-MR freeze-dryer (Labfreez Co., Ltd., Beijing, China) with a condenser temperature of <−55 °C, an ultimate vacuum pressure of 5 Pa, and a pump capacity of 2 L/s to obtain xerogels. The fractured sections of the xerogels were coated with gold/palladium (80/20) for SEM studies. For quantitative analysis of the microstructure of hydrogels, images were processed using MountainsLab software V11.0 (Digital Surf, Besançon, France).

### 4.7. Statistical Analysis

All measurements were carried out at least three times and expressed as the mean ± standard deviation; the obtained data were subjected to a one-way/two-way analysis of variance (ANOVA) using OriginPro 9.0 statistical software. Differences between the means were considered significant at *p* < 0.05. The significance of data points was subsequently determined by the Tukey test (*p* < 0.05).

## Figures and Tables

**Table 1:** Chemical composition of fish protein isolates obtained by two methods (under different conditions of alkaline dissolution of the protein) and protein-containing raw materials.

Sample	Moisture ω_M_ (%)	Total Nitrogen N_T_ (%)	Protein * P (%)	Mineral Substances ω_A_ (%)	Yield B (%)
FPI-1	5.2 ± 0.1 ^a^	15.0 ± 0.1 ^a^	93.8 ± 0.6 ^a^	0.7 ± 0.01 ^a^	10.4 ± 0.6 ^a^
FPI-2	5.9 ± 0.1 ^b^	15.0 ± 0.1 ^a^	93.8 ± 0.6 ^a^	0.4 ± 0.1 ^b^	14.4 ± 0.7 ^b^
Protein-containing raw material	80 ± 1 ^c^	3.0 ± 0.1 ^b^	18.8 ± 0.6 ^b^	0.9 ± 0.1 ^c^	–

* The protein mass fraction was calculated as P = N_T_ × 6.25 (6.25 is the conversion factor for the amount of nitrogen per protein). The nitrogen-to-protein conversion factor was selected in accordance with the EU Council Directive on Nutrition Labelling of Foods (90/496/EEC). Superscript letters indicate significant differences (*p* < 0.05) among the groups. Means ± standard deviation (*n* = 3).

**Table 2:** Water-holding capacity (WHC), fat holding capacity (FHC), emulsifying capacity (EC), emulsion activity index (EAI), and emulsion stability index (ESI) of fish protein isolates.

Isolates	WHC, g/g		FHC, g/g	EC, %	EAI, m^2^/g	ESI, min
	Water, pH 5.6–6.2	Buffer, pH 7.0				
FPI-1	6.2 ± 0.1 ^a^	8.6 ± 0.1 ^a^	3.0 ± 0.1 ^a^	66 ^a^	29.3 ^a^	100.8 ^a^
FPI-2	5.8 ± 0.1 ^b^	8.3 ± 0.1 ^b^	2.8 ± 0.1 ^a^	93 ^b^	31.6 ^b^	546.0 ^b^

Superscript letters indicate significant differences (*p* < 0.05) among the groups. Means ± standard deviation (*n* = 3).

**Table 4:** The effect of the recovery method on the glass transition and denaturation temperatures of two fish protein isolates (FPI-1and FPI-2).

Samples	Heating Rate (°C/min)	T_g_ (°C)	T_b_ (°C)	T_d_ (°C)	ΔH_g_ (J/g)
FPI-1	10		150.0 ± 0.8 ^a^	158.2 ± 1.0 ^a^	
	20	64.5 ± 0.5 ^a^	120.2 ± 0.1 ^b^	163.0 ± 1.9 ^b^	67.4 ± 1.2 ^a^
FPI-2	10		134.5 ± 0.8 ^c^	140.9 ± 2.0 ^c^	
	20	70.3 ± 0.9 ^b^	115.8 ± 0.2 ^d^	158.5 ± 0.6 ^d^	66.9 ± 1.0 ^a^

Superscript letters indicate significant differences (*p* < 0.05) among the groups. Means ± standard deviation (n = 3).

## Data Availability

Data are contained within the article.

## Abbreviations

The following abbreviations are used in this manuscript:

FPI	Fish protein isolate
FPI-1	Fish protein isolates with high-molecular mass
FPI-2	Fish protein isolates with low-molecular mass
ISP	Isoelectric solubilisation/precipitation
FT-IR	Fourier transform infrared
DSC	Differential scanning calorimetry
SEM	Scanning electron microscopy
